# A Narrative Review of College Meningococcal Vaccination Mandates Across the United States

**DOI:** 10.3390/vaccines13080784

**Published:** 2025-07-24

**Authors:** Jessica Presa, Eva Jodar, Monica Ochapa, Tim A. Mullenix, Sharon E. Barrett, Alejandro Cane

**Affiliations:** 1 Vaccines Medical Development and Scientific/Clinical Affairs, Pfizer Inc., 500 Arcola Rd, Collegeville, PA 19426, USA; alejandro.cane@pfizer.com; 2 Vaccines Health Economics and Outcomes Research, Pfizer Inc., 66 Hudson Blvd E, New York, NY 10001, USA; eva.jodarcasco@pfizer.com; 3 Public and Allied Health Program, School of Community Health and Policy, Portage Campus, Morgan State University, 1700 East Cold Spring Lane, Baltimore, MD 21251, USA; monica.ochapa@pfizer.com (M.O.); sharon.barrett@morgan.edu (S.E.B.); 4 Vaccines Medical and Scientific Affairs, Pfizer Inc., 500 Arcola Rd, Collegeville, PA 19426, USA; tim.mullenix@pfizer.com

**Keywords:** MenACWY, MenB, meningococcus, Neisseria meningitidis, university, vaccine

## Abstract

In the United States, adolescents and young adults between the ages of 16 and 23 have high rates of serogroup B meningococcal infections due to an elevated risk for those attending college. This review examines meningococcal vaccination requirements and recommendations for college students in the United States, with a focus on state-level mandates. National stakeholder resources, state legislatures, and official state Department of Health and Department of Education websites were analyzed for each state and Washington, DC. Overall, 26 states mandate MenACWY vaccination for college entry, whereas only 2 have specific requirements for MenB vaccination. Among the six states with the largest state university campus enrollments, half mandate MenACWY vaccination for college students, whereas none mandate MenB. By region, the Northeast had the highest percentage of states with a MenACWY requirement for college entry (77.8%) followed by the South (64.7%), Midwest (41.7%), and West (23.1%). Further research is needed to elucidate the relationship between state mandates and coverage to aid in optimizing meningococcal vaccination for college students.

## 1. Introduction

Invasive meningococcal disease (IMD) is a potentially fatal condition caused by infection with the Gram-negative bacterium *Neisseria meningitidis* [1]. The overall case fatality rate of IMD is high even when treated (up to 15% overall), and 10% to 20% of survivors experience long-term debilitating sequelae [1,2,3]. Humans represent the only known natural reservoir for *N. meningitidis* [1]. In most people, nasopharyngeal carriage of *N. meningitidis* is asymptomatic and leads to a protective antibody response; however, in a small proportion of individuals, these bacteria penetrate the mucosa and spread systemically via the bloodstream, resulting in IMD [1]. The meningococcal nasopharyngeal carriage rate peaks in early adulthood, with nearly one in four adolescents 19 years of age being asymptomatic carriers [4]. The high frequency of carriage has been associated with social behaviors common in this age group, such as intimate kissing and attending parties or bars [5].

The epidemiology of IMD remains unpredictable. Cases may occur sporadically or as part of larger outbreaks and are generally more common during the winter and early spring months [1,6]. The US Centers for Disease Control and Prevention reported case fatality rates for IMD in the United States of 9.6%, 10.4%, and 15.1% in 2019, 2020, and 2021, respectively [7,8,9]. IMD affects all age groups, with the highest rates of disease typically occurring in infants (age <1 year), young children (age 1–4 years), and adolescents/young adults (ages 16–23 years) [10]. However, the distribution of disease-causing serogroups varies by age [10]. In the United States, serogroup B (MenB) is a more common cause of IMD among infants, young children, and adolescents/young adults compared with older adults; conversely, disease caused by serogroups W and Y is generally more common among older adults compared with younger age groups [7,8,9,10]. Between 2015 and 2018 in the United States, 136 cases of IMD were reported among infants, with 68.4% caused by MenB; 223 cases were reported among adolescents and young adults, with 61.9% caused by MenB [10]. From 2019 to 2021, the number of IMD cases reported among infants dropped to 67, with 56.7% caused by MenB; however, in 2021, the percentage of IMD due to MenB peaked to 75%. During the same period, 91 cases of IMD were reported in adolescents, with 37.4% caused by Men B [7,8,9]. Additionally, an analysis of US disease surveillance data collected during 2014–2016 from individuals aged 18 to 24 years revealed a 3.5-fold higher risk of MenB disease in college students compared with noncollege students [11]. This increased risk is likely related to crowded living conditions among students carrying diverse *N. meningitidis* strains and social behaviors that favor transmission via respiratory secretions [1,11]. MenB was the cause of all IMD outbreaks at US colleges from 2011 to March 2019; these outbreaks affected 13 campuses, resulting in 50 cases and 2 deaths [12].

Until recently, the US Advisory Committee on Immunization Practices (ACIP) recommended two types of meningococcal vaccines for healthy adolescents [13]. Vaccination against meningococcal serogroups A/C/W/Y (MenACWY) is routinely recommended (that is, routinely offered during physician visits) for all individuals and consists of a primary dose at 11 to 12 years of age and a booster dose at 16 years [13]. MenB vaccination is recommended based on shared clinical decision-making (that is, based on an individual’s risk assessment and the healthcare provider’s clinical discretion) for adolescents and young adults aged 16 to 23 years (preferred age, 16–18 years) and consists of a two-dose primary series [13]. In October 2023, a pentavalent vaccine (Penbraya™; Pfizer Ireland Pharmaceuticals, Cork, Ireland) comprising licensed MenACWY-TT (Nimenrix^®^; Pfizer Inc., Sandwich, UK) and MenB-fHbp (Trumenba^®^, bivalent rLP2086; Pfizer Inc., Philadelphia, PA, USA) vaccines was approved by the US Food and Drug Administration for protection against the five main disease-causing serogroups (MenA/B/C/W/Y) in individuals aged 10 to 25 years [14,15]. In a first-in-human study, the MenABCWY vaccine was well tolerated and induced robust immune responses for all five serogroups among individuals 10 to 25 years old [16]. The ACIP has recently recommended that a single MenABCWY dose may be used when MenACWY and MenB vaccines are due for receipt at the same visit [17].

Although both MenACWY and MenB vaccination costs are reimbursed for adolescents, coverage in this age group varies considerably between the two vaccine types [18,19]. In 2022, US adolescents 17 years of age had an estimated vaccination coverage of 90.7% for ≥1 MenACWY dose and 60.8% for ≥2 MenACWY doses; by contrast, only 29.4% received ≥1 MenB dose and 11.9% received ≥2 MenB doses [18]. An analysis of 2017–2018 national immunization survey data for adolescents aged 17 years also revealed low coverage for ≥2 MenB doses and substantial regional variation in coverage levels [20]. Across the United States, fewer than half of the 17-year-olds who received ≥1 MenB dose also received a second dose [20]. Comparison among census regions revealed that MenB coverage was highest among 17-year-olds in the Northeast (18.3% for ≥1 dose, 9.3% for ≥2 doses) and lowest among those living in the South (14.6% and 6.3%, respectively) [20]. MenABCWY vaccines offer the potential to simplify the existing MenACWY and MenB vaccine schedules and in turn increase meningococcal vaccine uptake, in particular for MenB, ultimately averting a higher number of IMD cases [21,22]. A US population-based modeling scenario based on current MenACWY vaccine coverage rates estimated that administering two MenABCWY doses at 11 years of age and a further MenABCWY dose at 16 years of age may prevent a further 91 IMD cases over a 10-year period (compared with separate administration of MenACWY and MenB vaccines in the current schedule) [21].

This narrative review examines the landscape of meningococcal vaccination mandates for colleges in the United States. The prevalence and specifics of these mandates are explored, with a particular focus on identifying patterns across state-level policies, meningococcal disease occurrence (including outbreaks), and mandate implementation in states with large public universities.

## 2. Methods

Meningococcal vaccination requirements were obtained from national stakeholder resources (immunize.org and the American Society for Meningitis Prevention [previously called The Meningitis B Action Project]), the National Conference of State Legislatures, individual state legislature documents, and official state Department of Health and Department of Education websites. Required and recommended immunizations for incoming first-year students at public colleges and universities were examined for each US state and Washington, DC. To aid the presentation and discussion of results, the term “states” is used to broadly encompass states and Washington, DC, unless otherwise specified. For the purposes of this review, in discussion of state-level recommendations and requirements, “required” means that a student who has not received the mandated vaccine will be denied entry to college unless an exemption or waiver is allowed and “recommended” means that the vaccine is not mandated for college entry but is explicitly endorsed on the state’s college immunization page or a weblink to US Centers of Disease Control and Prevention guidelines for the vaccine was provided. In addition, for MenB vaccination, states with a more general recommendation for the adolescent/young adult age group are noted. Data from November 2023 were tabulated by vaccine type and state requirement or recommendation. MenB outbreak data were obtained from the American Society for Meningitis Prevention (previously called The Meningitis B Action Project; https://meningitisprevention.org, accessed on 15 November 2023), major news articles, college/university announcements, and the published literature. This article is based on previously conducted studies and does not contain any new studies with human participants or animals performed by any of the authors.

## 3. Results

**Figure 1:**
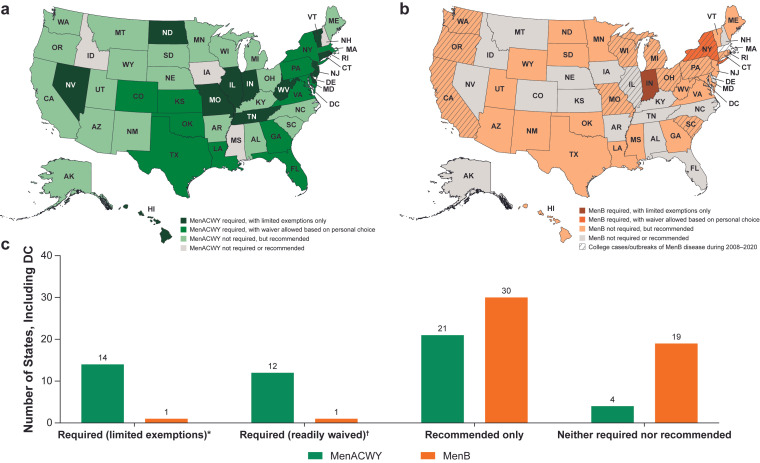
US map of state-level (**a**) MenACWY and (**b**) MenB vaccine requirements and recommendations for college entry and (**c**) summary of US state MenACWY and MenB vaccine requirements/recommendations for college entry [29]. MenACWY, meningococcal serogroups A, C, W, and Y; MenB, meningococcal serogroup B. * Exemptions allowed only if based on medical or religious grounds. ^†^ May be declined based on personal choice.

Currently, 26 states (including Washington, DC, USA) mandate MenACWY vaccination for college enrollment in public colleges and universities (Figure 1a,c); private colleges are not required to adhere to states’ requirements. Although 16 of those 26 states are required to provide students with information about meningococcal disease and the vaccine in addition to the mandate, only 12 of the 16 states allow students to decline vaccination by signing a waiver form after reviewing information about the vaccine. The remaining four states offer limited exemptions primarily focused on medical and religious reasons (Table 1). Of the 10 states that mandate MenACWY vaccination without information or waiver provision requirements, all permit for medical exemption. Nine of the ten states allow for exemption based on religious belief; some of these states also offer exemptions for students enrolled exclusively in distance/virtual learning. Overall, of the 26 states that require MenACWY vaccination for college entry, 14 do not allow students to decline vaccination based on personal choice alone. Among the 25 states that do not have a MenACWY vaccination requirement for college students, 21 recommend the vaccine (Figure 1a,c). There are few discrepancies between these findings and ones provided in national stakeholder resources, particularly Immunize.org [23]. Although the “Immunize.org” summary indicated a vaccine mandate in Ohio [23], the state-level requirements do not require MenACWY vaccination for college and university entry, just that students residing in on-campus housing must disclose their vaccination status [24,25]. Additional state-level MenACWY vaccine mandates in Washington, DC [26,27], and West Virginia [28] were identified that are not included in the Immunize.org summary.

Texas, Florida, Ohio, Arizona, Illinois, and Minnesota had the largest single public university campuses by enrollment in the 2022–2023 academic year, with student populations in excess of 50,000 [131,132,133,134,135,136,137,138]. Notably, only Illinois [23,26,55] has a MenACWY vaccine mandate that cannot be waived (Table 1). Florida [26,45,46] and Texas [26,117] also have MenACWY vaccine mandates; however, these can be declined after reading the provided vaccine information. Arizona [23,33,34], Ohio [24,25], and Minnesota [23,73,74] do not require MenACWY vaccination but do have a state-level MenACWY recommendation. Table 1 provides further details of the state-level MenACWY vaccination requirements and recommendations for all 50 states and Washington, DC.

Indiana [58] and New York [26,95,96] have state-level MenB vaccination requirements for college students (Table 1). Indiana mandates both MenACWY and MenB vaccination for students aged ≤23 years [26,56,57,58]. In New York, college entry requires proof of ≥1 dose of MenACWY vaccine within the last 5 years, a complete MenB vaccine series, or a completed response form declining meningococcal vaccination or indicating meningococcal vaccination will be obtained within 30 days [26,95,96]. Among the 49 states that do not have any state-level MenB vaccine mandate, 30 recommend MenB vaccination for college students or college-age individuals (Figure 1b,c). Considering college-level mandates as of May 2023, 16 states had at least some colleges that required MenB vaccination for entry and a further 19 states had at least some colleges that recommended MenB vaccination to their students (Table 2) [29].

Among all the states and the District of Columbia, 14 had MenB cases or outbreaks on college campuses in the 12-year period between 2008 and 2020 (Table 2 and Table 3) [29]. Of these, New York is the only state with a state-level MenB mandate for college students [96], while 11 other states have a state-level recommendation for MenB vaccination of college students or college-age individuals (Table 1) [29].

Among states with the largest public university campuses by enrollment in 2022–2023 (Texas, Florida, Ohio, Arizona, Illinois, and Minnesota) [131,132,133,134,135,136,137,138], only Ohio and Illinois experienced campus MenB cases or outbreaks between 2008 and 2020 (Table 2) [29]. Texas [118,119], Ohio [24,25], Arizona [33,34], and Minnesota [73,74] recommend but do not require MenB vaccination at the state level. In contrast, Florida [26,45,46] and Illinois [23,26,55] have no state-level MenB vaccination requirements or recommendations (Table 1); however, as of May 2023, Florida has only one college and Illinois has five colleges that recommend MenB vaccination (Table 2) [29].

## 4. Discussion

**Figure A1:**
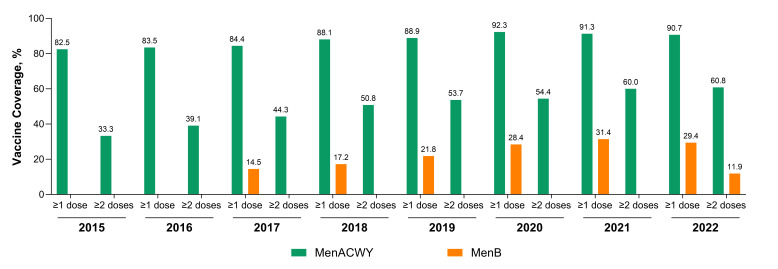
MenACWY and MenB * vaccine coverage in US adolescents 17 years of age from 2015 to 2022 [7,18,149,150,151,152,153]. MACWY, meningococcal serogroups A, C, W, and Y; MenB, meningococcal serogroup B. * MenB vaccination coverage for ≥1 and ≥2 doses was not estimated until 2017 and 2022, respectively.

Available information indicates that, as of November 2023, 26 US states had mandates in place for the MenACWY vaccination of college students, whereas only 2 states had a MenB vaccination requirement for college entry. Notably, all college outbreaks of meningococcal disease from 2011 to March 2019 were caused by MenB. These outbreaks affected 13 campuses, resulting in 50 cases and 2 deaths among a population of 253,000 students who were at risk of meningococcal disease during this time [12]. Annual US meningococcal disease surveillance data for 2019–2021 show a continuing predominance of serogroup B disease in colleges, with MenB accounting for 63% to 67% of cases with a known serogroup each year among college students aged 18 to 24 years [7,8,9]. Of further concern, although 2022 national survey data indicate that US MenACWY coverage in 17-year-olds was 90.7% for ≥1 MenACWY dose and 60.8% for ≥2 MenACWY doses, MenB vaccine coverage for ≥1 dose reached only a third of MenACWY coverage levels and was lower than 20% for ≥2 doses (Figure A1) [18]. Consequently, MenB vaccine mandates and recommendations for college students are particularly important to ensure that individuals are adequately informed regarding their risk of MenB disease and the availability of protective vaccines.

With the aim of protecting health and reducing outbreaks for students attending college, the American College Health Association (ACHA) provides guidelines on immunizations consistent with ACIP recommendations [140]. The ACHA recommends a primary dose of MenACWY vaccine at 11 to 12 years of age and a booster dose at 16 years; these guidelines further indicate that for colleges with meningococcal vaccine requirements for enrollment or residing on campus, students ≤21 years of age should provide proof of having received a MenACWY dose at age 16 years or older [140]. The ACHA also recommends routine MenB vaccination as a two- or three-dose primary series for all individuals at increased risk of disease or for adolescents/young adults aged 16 to 23 years (preferred age, 16–18 years) who are not at increased risk, as recommended on the basis of shared clinical decision-making [140]. Despite ACIP’s introduction of MenB vaccination recommendations in 2015 [141] and the ongoing persistence of MenB disease among college students, only Indiana and New York have any state-level MenB vaccination requirement for college entry [58,96].

Statewide mandates for MenB vaccination would help prevent outbreaks, which are challenging to control and both economically and socially costly [142]. Economic modeling based on the 2015 IMD outbreak at the University of Oregon and the 2016 IMD outbreak at Oregon State University conservatively estimate USD 12.3 million in costs associated with each outbreak response [143]. Compared with routine preventive MenB vaccination, providing reactive vaccination increases the economic burden by an estimated USD 8 million in direct costs associated with mass vaccination clinics [142,143]. Response strategies to MenB outbreaks were catalogued in a review of college MenB outbreaks in the United States between 2013 and 2018 that showed that 10 states with college outbreaks reactively deployed MenB vaccines [144]. Response measures varied and included the use of mass vaccination campaigns, making the vaccine available through the university’s health clinic, and encouraging students to get vaccinated through pharmacies and healthcare providers. Notably, Oregon State University implemented a college-level MenB vaccine requirement [144].

### 4.1. Patterns of Vaccination Requirements and Coverage

This review demonstrates that the number of states requiring a MenACWY or MenB vaccine for college entry has remained relatively constant. A review of state-level college mandates up to 2018 showed that 24 states required MenACWY vaccination and that 1 state required MenB vaccination [145], and the current study shows only a slight increase. As of November 2023, of the 26 states with mandates for meningococcal vaccines, 24 (92.3%) required the MenACWY vaccine only, 1 required either MenACWY or MenB vaccine, 1 required both vaccines, and none required the MenB vaccine only. Regional differences in MenACWY requirements at the state level were also observed: 77.8% of states in the Northeast, 64.7% in the South, 41.7% in the Midwest, and 23.1% in the West required MenACWY for college entry. Notably, among the six states with the largest US college campuses by 2022–2023 enrollment, only one (Illinois [23,26,55]) has a state-level college mandate for the MenACWY vaccine that cannot be readily waived and none have a MenB vaccine mandate.

The results of this review are similar to a 2017 survey of 352 colleges throughout the United States that determined that 52.8% required and 42.1% recommended meningococcal vaccines [146]. Among the colleges that required vaccination, 69.9% required only the MenACWY vaccine, 3.8% required both the MenACWY and MenB vaccines, and none required only the MenB vaccine; the remaining 26.3% did not specifically define which meningococcal vaccine(s) were required [146]. By region, the Northeast had the highest percentage of colleges with meningococcal vaccine requirements (72.0% of respondents to the 2017 survey), followed by the South (59.2%), Midwest (42.2%), and West (23.1%) [146]. As college size decreased, a requirement for meningococcal vaccination was more common, with the largest colleges (≥20,000 undergraduate students) more often recommending (approximately 54%) rather than requiring (approximately 37%) meningococcal vaccination. In contrast, approximately 75% of small colleges (<1000 undergraduate students) surveyed required meningococcal vaccination [146].

Studies investigating patterns of MenACWY vaccine coverage in US adolescents have also observed regional disparities similar to those observed in this review with college entry requirements [147,148]. Among 17-year-olds in the National Immunization Survey—Teen 2017, up-to-date MenACWY coverage was significantly lower in the Midwest compared with the Northeastern (adjusted odds ratio [aOR], 0.72 [95% CI: 0.52, 0.99), Southern (aOR, 0.36 [95% CI: 0.27, 0.49) and Western (aOR, 0.35 [95% CI: 0.28, 0.54) census regions [147]. The same study also identified significantly higher median up-to-date MenACWY coverage rates among states with a one-dose or two-dose requirement for school entry (54.1% and 63.6%, respectively) compared with no requirement (41.5%; *p* = 0.001); interestingly, neither the type of nor ease of obtaining exemptions to state-level requirements were found to be associated with a significant difference in MenACWY coverage [147].

A retrospective analysis of the Commercial Claims and Encounters MarketScan database from 2011 to 2016 demonstrated significant disparity in uptake of ≥1 MenACWY dose in younger (observed 10.5–13 years of age to coincide with primary MenACWY vaccination; 71.7% uptake) versus older (observed 15.5–18 years of age to coincide with booster MenACWY vaccination; 48.9% uptake) US adolescents [148]. Regression modeling indicated that the lower uptake of MenACWY in older adolescents was largely due to the receipt of fewer non-MenACWY vaccines, attending fewer preventive healthcare visits, and interaction with nonpediatric healthcare providers. This study also evaluated uptake across regions, which revealed similar rates for ≥1 MenACWY dose in the younger adolescents (Northeast, 72.0%; North Central [equivalent to the census Midwest region], 73.7%; South, 72.4%; and West, 67.3%). However, uptake rates in the older adolescents showed wider variation (Northeast, 59.4%; North Central, 50.0%; South, 47.0%; and West, 39.6%) [148]. The higher MenACWY uptake in the Northeast was attributed to a greater number of preventive care and well-child visits. Based on the findings of the current review, it is possible that a higher frequency of states requiring MenACWY for college entry in the Northeast may have also contributed.

### 4.2. Recommendations

Although this review offers valuable insights into the current landscape of meningococcal vaccination mandates for college students, it is important to acknowledge its limitations because the findings are contingent upon the current policy landscape, which is susceptible to change. To maintain the accuracy and relevance of this information, several avenues for future research and knowledge dissemination will be proposed.

Firstly, the dynamic nature of vaccination mandates renders a static review a mere snapshot in time. State and national policies are in constant flux, necessitating a system for continuous monitoring. Developing an interactive database, a regularly updated website, or a collaborative network of public health institutions could provide invaluable real-time data, allowing for timely revisions and a more nuanced understanding of the evolving policy landscape.

Secondly, although the historical and ongoing focus on MenACWY mandates remains crucial, the landscape is shifting. The emergence of the MenB strain as a significant threat to college students calls for a wider understanding beyond MenACWY-centric policies [144]. Disease due to serogroups contained in MenACWY has been a well-established public health concern for decades, whereas serogroup B disease has only emerged as a significant threat in more recent years [7,8,9,10]. This means many states have yet to consider or implement specific MenB vaccination mandates for college students and, as a result, the understanding of the policy landscape in this area remains incomplete. Future research should actively track and map existing MenB mandates for college students across different states to provide a vital baseline for understanding current policy trends and identifying potential gaps. Additionally, reevaluating pre-2015 waiver forms and resources with the lens of MenB vaccine availability would offer valuable insights into historical considerations and potential policy blind spots, and therefore inform the development of more comprehensive and effective policies for the future.

Finally, the direct relationship between state mandates and vaccination coverage among college students remains unclear. Future research should elucidate this critical connection through longitudinal studies that include assessments on the impact of mandates on coverage rates over time, comparisons between mandated and non-mandated states, and investigations into the role of campus-specific factors, such as vaccine information campaigns and enforcement mechanisms. Defining the impact of these variables could also provide answers on the factors influencing the state-level adoption of MenB vaccination mandates, elucidate the economic and social impacts of IMD outbreaks on college campuses, and illuminate the ethical considerations surrounding mandatory vaccination policies. A clearer understanding of these relationships would inform the effectiveness of mandates and guide the development of more targeted vaccination strategies to optimize adolescent and young adult coverage. Additionally, gathering perspectives from diverse stakeholders, including public health officials, college administrators, and students, could offer valuable insights for shaping future policy directions. With an understanding of these multifaceted aspects, work could be carried out to bridge the current gap and establish more comprehensive meningococcal vaccination policies that effectively safeguard the health and well-being of US college students.

## 5. Conclusions

Across the United States, MenACWY vaccination for college entry is mandated in half of all states, with the remaining states predominantly recommending this crucial public health measure. This widespread concurrence underscores the importance of protecting college students from meningococcal disease. Notably, a correlation exists between states mandating MenACWY vaccination and those exhibiting higher uptake rates among older adolescents, especially in the Northeast region, suggesting the potential effectiveness of mandatory policies in boosting vaccination coverage.

However, a concerning disparity emerges when considering MenB vaccination. Despite MenB predominance in college-related IMD outbreaks and cases since 2008 and the 2015 ACIP recommendation for the MenB vaccination, only two states currently mandate MenB vaccination for college students. This discrepancy between existing recommendations and implemented policies highlights the need for further policy action to address this gap and ensure comprehensive meningococcal disease prevention strategies are in place for this vulnerable population. Implementation of information campaigns regarding meningococcal disease and vaccination, particularly in states and at colleges without meningococcal vaccination requirements, would also be a beneficial strategy to help prevent meningococcal disease among adolescents and college students.

## Figures and Tables

**Table 1:** Summary of state-level college MenACWY and MenB vaccine mandates and recommendations.

State	MenACWY Required	Students Given Information About MenACWY Vaccine; Students May Decline Vaccination After Review	MenACWY Recommended	MenB Required	MenB Recommended	Exemptions
Alabama [23,30]	NO	NO; N/A	YES *	NO	NO	N/A
Alaska [23,31,32]	NO	YES; YES	YES	NO	NO	N/A
Arizona [23,33,34]	NO	NO; N/A	YES	NO	YES	N/A
Arkansas [23,35,36,37]	NO	NO; N/A	YES	NO	NO	N/A
California [23,38]	NO	YES; YES	YES	NO	YES	N/A
Colorado [26,39,40,41]	YES for students residing in student housing	YES; YES	N/A	NO	NO	N/A
Connecticut [26,42,43]	YES for students residing in on-campus housing	YES; NO (compulsory for students in on-campus housing)	N/A	NO	YES	Medical and religious
Delaware [23,26,44]	YES required within 5 years before enrollment	YES; NO (compulsory for students who wish to enroll in post-secondary schools)	N/A	NO	NO	Students may submit a written request to the postsecondary educational institution for exemption
Florida [26,45,46]	YES for students residing in on-campus housing	YES; YES	N/A	NO	NO	N/A
Georgia [23,26,47,48]	YES for students residing in campus housing or sorority/fraternity houses	YES; YES	N/A	NO	YES	N/A
Hawaii [26,49,50,51]	YES for first-year students residing in campus housing	NO; N/A	N/A	NO	YES *	Medical and religious; religious exemptions to specific immunizing agents will not be granted
Idaho [23,52,53,54]	NO	YES; YES	NO	NO	NO	N/A
Illinois [23,26,55]	YES for incoming students aged <22 years	NO; N/A	N/A	NO	NO	Medical and religious
Indiana [26,56,57,58]	YES for students aged ≤23 years	NO; N/A	N/A	YES for students aged ≤23 years	N/A	Medical and religious
Iowa [23,59,60]	NO	YES; YES	NO	NO	NO	N/A
Kansas [26,41,61]	YES for students residing in student housing	YES; YES	N/A	NO	NO	N/A
Kentucky [23,62]	NO	NO; N/A	YES	NO	NO	N/A
Louisiana [26,41,63,64]	YES for all on-campus students	YES; YES	N/A	NO	YES	Medical, religious, and personal; requires review of information by individual seeking an exemption for religious or personal reasons
Maine [23,65,66,67]	NO	NO; N/A	YES for freshman residing in a dormitory	NO	YES *	N/A
Maryland [26,68,69]	YES for students residing in on-campus student housing	YES; YES	N/A	NO	YES *	N/A
Massachusetts [26,70,71]	YES for full-time students aged <22 years residing in dormitory or with living arrangements licensed/approved by the school	YES; YES	N/A	NO	NO	Medical, religious, and personal; requires review of information by individual seeking an exemption for religious or personal reasons
Michigan [23,72]	NO	NO; N/A	YES for freshman residing in a dormitory	NO	YES *	N/A
Minnesota [23,73,74]	NO	YES; YES	YES for freshman residing in a dormitory	NO	YES *	N/A
Mississippi [23,75,76,77]	NO	NO; N/A	NO	NO	YES *	N/A
Missouri [26,41,78,79,80]	YES for students residing in campus housing or sorority/fraternity houses	YES; NO (compulsory for students residing in campus housing or sorority/fraternity houses)	N/A	NO	YES *	Medical and religious
Montana [23,81,82]	NO	NO; N/A	YES *	NO	NO	N/A
Nebraska [23,83,84]	NO	YES; YES	YES for college freshman, in particular those residing in a dormitory and for students who did not receive dose 2 at age 16 years	NO	NO	N/A
Nevada [26,85]	YES for all on-campus freshman aged <23 years	NO; N/A	N/A	NO	NO	Medical and religious; students enrolled in distance education only
New Hampshire [23,86,87]	NO	NO; N/A	NO	NO	NO	N/A
New Jersey [26,88,89,90,91,92]	YES	YES; NO	N/A	NO ^†^	YES	Medical and religious
New Mexico [23,93,94]	NO	NO; N/A	YES for freshman residing in student housing if not previously vaccinated at age 16 years or older	NO	YES *	N/A
New York [26,95,96]	YES ^‡^ for students enrolled for ≥6 h per semester or ≥4 h per quarter	YES; YES ^‡^	N/A	YES ^‡^	N/A	N/A
North Carolina [23,97,98]	NO	NO; N/A	YES for freshman residing in a dormitory	NO	NO	N/A
North Dakota [26,99,100]	YES for students aged <22 years	NO; N/A	N/A	NO	YES *	Medical and religious; exemptions also provided to students enrolled only in distance learning, continuing education, noncredit, or off-campus courses and for students attending camps, workshops, courses, or programs contracted to a third party
Ohio [24,25]	NO (but students residing in on-campus student housing must disclose meningitis vaccination status)	NO; N/A ^§^	YES	NO	YES	N/A
Oklahoma [26,41,94,101,102]	YES for incoming students residing in on-campus housing	YES; YES	N/A	NO	YES *	N/A
Oregon [23,103,104]	NO	YES; YES ^¶^	YES	NO	YES	N/A
Pennsylvania [26,41,105,106,107]	YES for students residing in a dormitory or housing unit	YES; YES	N/A	NO	YES *	Medical and religious; exemptions also provide for “other reasons” if the institution provides detailed information on the risks associated with meningococcal disease and the availability and effectiveness of any vaccine
Rhode Island [26,77,108,109]	YES for full-time previously unvaccinated students aged <22 years residing in dormitory or with living arrangements approved by the school	NO; N/A	N/A	NO	YES *	Medical and religious (214-30-05-3.6.3)
South Carolina [23,110,111,112]	NO	YES; YES	YES *	NO	YES *	N/A
South Dakota [23,113]	NO	NO; N/A	YES	NO	YES	N/A
Tennessee [26,114,115,116]	YES for incoming students aged <22 years residing in on-campus housing	NO; N/A	N/A	NO	NO	Medical and religious
Texas [26,117,118,119]	YES for incoming on-campus students aged <22 years	YES; YES	N/A	NO	YES *	Medical, religious, and personal belief
Utah [23,94,120]	NO	NO; N/A	YES for freshman residing in a dormitory who had not been vaccinated at age ≥16 years	NO	YES *	N/A
Vermont [26,121,122,123]	YES for freshman aged <22 years residing in a dormitory who received dose 1 before age 16 years	NO; N/A	N/A	NO	YES *	Medical and religious
Virginia [26,94,124,125]	YES for incoming full-time students	YES; YES	N/A	NO	YES *	Medical and religious; also, any student may submit a written waiver after reviewing provided information
Washington [23,94,126,127]	NO	YES; YES **	YES for freshman residing in a dormitory who had not been vaccinated at age ≥16 years	NO	YES *	N/A
Washington, DC [26,27]	YES for students residing in school housing and athletes	NO; N/A	N/A	NO	NO	Medical and religious
West Virginia [28]	YES	NO; N/A	N/A	NO	YES for students aged 16–23 years who are not at increased risk of disease or for any student at risk due to anatomical or functional asplenia, persistent complement deficiency, complement inhibitor use, or a meningococcal B outbreak	Medical; students attending in an all-virtual capacity may also qualify
Wisconsin [23,94,128,129]	NO	YES; YES ^††^	YES for freshman residing in a dormitory who had not been vaccinated at age ≥16 years	NO	YES *	N/A
Wyoming [23,94,130]	NO	NO; N/A	YES for freshman residing in a dormitory who had not been vaccinated at age ≥16 years	NO	YES *	N/A

MenACWY, meningococcal serogroups A, C, W, and Y; MenB, meningococcal serogroup B; N/A, not applicable. * Recommendation based on general age range. No evidence of a college-specific recommendation. ^†^ In accordance with the recommendations of the US Advisory Committee on Immunization Practices, MenB vaccine may be required for certain individuals with increased risk for meningococcal disease. ^‡^ Proof of ≥1 dose of MenACWY vaccine within the last 5 years, a complete MenB vaccine series, or a completed form either declining meningococcal vaccination or indicating that meningococcal vaccination will be obtained within 30 days is required. ^§^ The Ohio Department of Health shall make available information about meningococcal disease and vaccines that schools may use. Students residing in on-campus housing must disclose vaccination status. ^¶^ Schools that provide student housing are required to provide information about vaccine-preventable diseases to students enrolling and registering for the first time. ** Public and private colleges (excluding community and technical colleges) that offer on-campus or group housing are required to provide information about meningococcal disease to all incoming students. Community and technical colleges are required to provide the information only to those students who are offered on-campus or group housing. ^††^ School must require students residing in a dormitory (or if a minor, the parent or legal guardian) to affirm receipt of meningococcal vaccine information, state whether the student has received the vaccine, and provide the vaccination date, if applicable.

**Table 2:** Summary of college-level MenB vaccine requirements/recommendations and MenB cases/outbreaks during 2008–2020 [29].

State	Colleges with MenB Required	Colleges with MenB Recommended	College Cases/ Outbreaks of MenB ^‡^
Alabama	NO	NO	NO
Alaska	NO	NO	NO
Arizona	NO	YES	NO
Arkansas	NO	YES	NO
California	YES	YES	YES
Colorado	NO	YES	NO
Connecticut	NO	YES	YES
Delaware	YES	YES	NO
Florida	YES	YES	NO
Georgia	YES	YES	NO
Hawaii	NO	NO	NO
Idaho	YES	NO	NO
Illinois	NO	YES	YES
Indiana	YES (MenB mandated at state level)	YES	NO
Iowa	YES	YES	NO
Kansas	NO	NO	NO
Kentucky	NO	YES	NO
Louisiana	NO	NO	NO
Maine	NO	NO	NO
Maryland	NO	YES	NO
Massachusetts	NO	YES	YES
Michigan	YES	YES	YES
Minnesota	NO	YES	NO
Mississippi	YES	NO	NO
Missouri	NO	YES	YES
Montana	NO	NO	NO
Nebraska	NO	NO	NO
Nevada	NO	NO	NO
New Hampshire	YES	YES	NO
New Jersey	YES	YES	YES
New Mexico	NO	NO	NO
New York	YES (MenB mandated at state level *)	YES	YES
North Carolina	NO	NO	NO
North Dakota	NO	NO	NO
Ohio	YES	YES	YES
Oklahoma	NO	NO	NO
Oregon	NO	YES	YES
Pennsylvania	YES	YES	YES
Rhode Island	NO	YES	YES
South Carolina	YES	YES	NO ^†^
South Dakota	NO	YES	NO
Tennessee	NO	YES	NO
Texas	NO	YES	NO
Utah	NO	YES	NO
Vermont	NO	YES	NO
Virginia	YES	YES	NO
Washington	NO	YES	NO
Washington, DC	NO	NO	YES
West Virginia	NO	NO	NO
Wisconsin	NO	YES	YES
Wyoming	NO	NO	NO

MenACWY, meningococcal serogroups A, C, W, and Y; MenB, meningococcal serogroup B. * Proof of ≥1 dose of MenACWY vaccine within the last 5 years, a complete MenB vaccine series, or a completed response form declining meningococcal vaccination or indicating that meningococcal vaccination will be obtained within 30 days is required. ^†^ Serogroup of the case recorded in Meningitis Action Project at Clemson University in 2009 was unverifiable. This has been excluded from our analysis. ^‡^ Schools with past MenB cases in 2008–2020; data compiled from publicly available campus immunization forms.

**Table 3:** Summary of state MenB vaccination requirements for college students in states with campus MenB outbreaks or cases during 2008–2020 * [12,29,139].

State	Outbreaks of MenB Disease	Single Cases of MenB Disease	State-Level MenB Vaccine Requirement/ Recommendation for College Students
California	2014: University of California, Santa Barbara (5 cases) 2016: Santa Clara University (3 cases) 2018: San Diego State University (3 cases)	2014: Palomar College 2014: San Diego State University (resulted in death) 2015: University of California, Davis 2017: California Polytechnic State University 2017: Santa Barbara City College	Recommended
Connecticut		2018: Central Connecticut State University	Recommended
Illinois		2017: University of Illinois at Urbana, Champaign	None
Massachusetts	2017: University of Massachusetts Amherst and Five College Consortium (3 cases)	2018: Smith College	None
Michigan		2013: Kalamazoo College (resulted in death)	Recommended ^†^
Missouri		2015: Missouri University	Recommended ^†^
New Jersey	2013–2014: Princeton University (9 cases/1 death) 2016: Rutgers University (2 cases)		Recommended
New York	2008: Cornell University (2 cases) 2019: Columbia University School of International and Public Affairs (2 cases)	2008: State University College, Oswego (resulted in death) 2018: Colgate University 2018: Syracuse University	Required ^‡^
Ohio	2008–2010: Ohio University (10 cases/1 death)		Recommended
Oregon	2015: University of Oregon (7 cases/1 death) 2016: Oregon State University (5 cases)		Recommended
Pennsylvania	2009: University of Pennsylvania (3 cases) 2011: Lehigh University (2 cases) 2017: Bucknell University (2 cases)	2014: Drexel University ^§^ (resulted in death) 2017: Kutztown University 2018: Pennsylvania State University	Recommended ^†^
Rhode Island	2015: Providence College (2 cases)		Recommended ^†^
Washington, DC		2014: Georgetown University (resulted in death)	None
Wisconsin	2016: University of Wisconsin, Madison (3 cases)	2013: University of Wisconsin, Madison (resulted in death)	Recommended ^†^

* References were cross-checked and confirmed via internet resources when in disagreement. Major news articles and college/university announcements were used for verification of status as case or outbreak. Cases and outbreaks unconfirmed via additional resources were removed from this table. ^†^ Recommendation based on general age range (no evidence of a college-specific recommendation). ^‡^ Proof of ≥1 dose of MenACWY vaccine within the last 5 years, a complete MenB vaccine series, or a completed form either declining meningococcal vaccination or indicating that meningococcal vaccination will be obtained with 30 days is required. ^§^ Officially classified as a Princeton University outbreak case.

## Data Availability

No new data were created or analyzed in this study. Data sharing is not applicable to this article.

## Abbreviations

The following abbreviations are used in this manuscript:

ACHA	American College Health Association
ACIP	Advisory Committee on Immunization Practices
IMD	Invasive meningococcal disease
MenACWY	Meningococcal serogroups A/C/W/Y
MenB	Meningococcal serogroup B
